# Operando Characterization of Uranium Dendrite Growth in High‐Temperature Molten Salt Electrochemistry by Synchrotron X‐Ray Tomography and Diffraction

**DOI:** 10.1002/advs.202502345

**Published:** 2025-06-11

**Authors:** Kui Liu, Tan Tan, Yuke Zhong, Yafei Wang, Tao Bo, Zimei Bai, Shanfeng Wang, Kai Zhang, Wanxia Huang, Jianrong Zeng, Weiqun Shi

**Affiliations:** ^1^ Sino‐French Institute of Nuclear Engineering and Technology Sun Yat‐sen University Zhuhai 519000 China; ^2^ Institute of High Energy Physics Chinese Academy of Sciences Beijing 100049 China; ^3^ Institute of Nuclear Fuel cycle and Materials School of Mechanical Engineering Shanghai Jiao Tong University Shanghai 200240 China; ^4^ Engineering Laboratory of Advanced Energy Materials Ningbo Institute of Materials Technology and Engineering Chinese Academy of Sciences Ningbo 315201 China; ^5^ University of Chinese Academy of Sciences Beijing 101407 China; ^6^ Shanghai Synchrotron Radiation Facility Shanghai Advanced Research Institute Chinese Academy of Sciences Shanghai 201204 P. R. China; ^7^ Shanghai Institute of Applied Physics Chinese Academy of Sciences Shanghai 201800 P. R. China

**Keywords:** dendrite growth, molten salt electrochemistry, operando SR‐Î¼CT and HEXRD, pyroprocessing, uranium

## Abstract

In this study, an innovative operando characterization methodology utilizing synchrotron radiation X‐ray micro‐computed tomography (SR‐µCT) and high‐energy X‐ray diffraction (HEXRD) to investigate dendritic electrodeposition in high‐temperature molten salt (HTMS) electrochemistry is presented. This approach enables the in‐situ visualization and quantification of uranium dendrite growth and ion diffusion dynamics during the electrochemical reaction of LiCl‐KCl‐UCl_3_ molten salt. Through 3D reconstruction of uranium dendrite imaging and multiphysics simulations of the uranium electrolysis process, the underlying mechanisms of uranium dendrite formation are elucidated. Additionally, in situ HEXRD analysis of electrodeposited uranium reveals that the dendritic growth morphology is intrinsically linked to its crystal structure and orientation. This research not only advances the understanding of uranium dendrite growth and evolution but also establishes a foundational framework for the development of effective dendrite suppression strategies. These findings contribute significantly to the field of HTMS electrochemistry and have potential implications for the design of advanced electrochemical systems.

## Graphical abstract

An operando methodology combining synchrotron X‐ray micro‐computed tomography (SR‐µCT) and high‐energy X‐ray diffraction (HEXRD) investigates uranium dendrite electrodeposition in LiCl‐KCl‐UCl^3^ molten salt. This enabled in‐situ visualization of dendrite growth and ion diffusion dynamics. Utilizing 3D reconstructions and simulations, formation mechanisms are elucidated, while HEXRD reveals intrinsic links between growth morphology and crystal orientation, providing a basis for future dendrite suppression strategies.

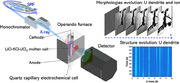

## 1 Introduction

HTMS electrochemistry plays an important role in a wide range of applications including metallurgy,^[^ ^1^ ^]^ spent nuclear fuel recycling,^[^ ^2^ ^]^ CO_2_ capture,^[^ ^3^ ^]^ etc. Among these applications, electrolysis is generally performed to extract or separate the element of interest from the electrode. The electrolysis in molten salt often comes with the growth of elongated, branched structures, commonly referred to as “dendrites,” of the electrodeposited products. For instance, the electrometallurgy of refractory metals,^[^ ^[Link]^, ^[Link]^ ^]^ the electro‐upcycling of metal scrap aluminum,^[^ ^4^ ^]^ and the spent nuclear fuel recycling of uranium.^[^ ^5^ ^]^ The growth of dendrites in electrolysis influences the electrochemical reaction stability, reduces the electrolysis efficiency, and imposes safety issues that impede the development of these HTMS applications. In Li‐metal batteries,^[^ ^6^ ^]^ the dendrite growth issue also exists, and has received a great deal of attention. However, the study of dendrite growth for molten salt applications was rarely carried out due to the high temperature and highly corrosivity environment.

Developing an effective method to monitor in situ the generation and growth of dendrite electrodeposition in HTMS is crucial to understanding the evolution mechanism of the morphology and structure of dendrites. This is essential for developing effective strategies to prevent dendrite growth and taking necessary precautions. In recent years, the development of in situ real‐time detection techniques has attracted great attention in numerous fields of research. For instance, by using the X‐ray computed tomography (CT), the battery industry has made great strides in understanding the mechanism and kinetics of dendrite growth of Li^[^ ^6b^ ^6^, ^7^ ^]^ and Zn^[^ ^8^ ^]^ under real‐time conditions at room temperature. For a high‐temperature system, Chen‐Wiegart et al.^[^ ^9^ ^]^ presented an in situ real‐time 3D X‐ray nano‐tomography imaging to directly visualize and quantify the dealloying process of Ni‐Cr alloy in HTMS. Wang et al.^[^ ^10^ ^]^ also developed a radionuclide tracing‐based in situ corrosion and mass transport monitoring method for the corrosion study of 316L stainless steel in a molten salt natural circulation loop.

For the electrochemistry study in HTMS, Jiao et al.^[^ ^11^ ^]^ recently designed a high‐temperature electrochemical facility equipped with lab‐based X‐ray CT and have achieved the nondestructive and quantitative 3D imaging for the titanium electrorefining process. However, the spatial resolution of the 3D tomograms generated by the equipped lab‐based X‐ray CT is only at 6–10 µm per voxel, which is too low to capture the generation and growth of the dendrite electrodeposition in HTMS electrochemistry. Currently, it is still critically challenging to perform high‐resolution operando analysis for the electrolysis process in HTMS. Unlike lab‐based X‐ray CT, SR‐µCT equipped with high brightness and collimation of the X‐ray source can provide 3D tomograms with high spatial and temporal resolutions. In addition, the synchrotron HEXRD can track the structures and phase transitions of the electrodeposited products in real‐time during the HTMS electrodeposition process. Therefore, coupling synchrotron µCT and XRD techniques in an HTMS electrochemical testing cell is promising to obtain a high‐resolution operando characterization on the morphology and structure evolution of the dendrite electrodeposition in HTMS.

In this study, we developed an integrated system coupling SR‐µCT and HEXRD techniques to carry out the in situ detection of the electrochemical reaction process in HTMS using a self‐designed quartz capillary electrochemical cell (QCEC). As the main fuel material of clean nuclear energy, uranium, the study of its dendrite growth in HTMS electrochemistry for the application of pyroprocessing of spent nuclear fuel was focused on. Using the SR‐µCT, the uranium dendrite growth and ion diffusion during the electrochemical reaction process were visualized and quantified. Combining with the multi‐physics simulations, the uranium dendrite growth mechanism was disclosed from the view of ion diffusion. The crystal lattice evolution of uranium dendrite was also deciphered through HEXRD, and the preference of dendrite growth was explained by the crystal plane orientation. The present study represents a pioneering high‐resolution work for the in‐situ analysis of electrochemical reaction mechanism and evolution of uranium dendrite growth in HTMS, which is an important step toward inhibiting the dendrite electrodeposition issue in molten salt electrolysis.

## 2 Results

### 2.1 Operando SR‐µCT and HEXRD

**Figure 1:**
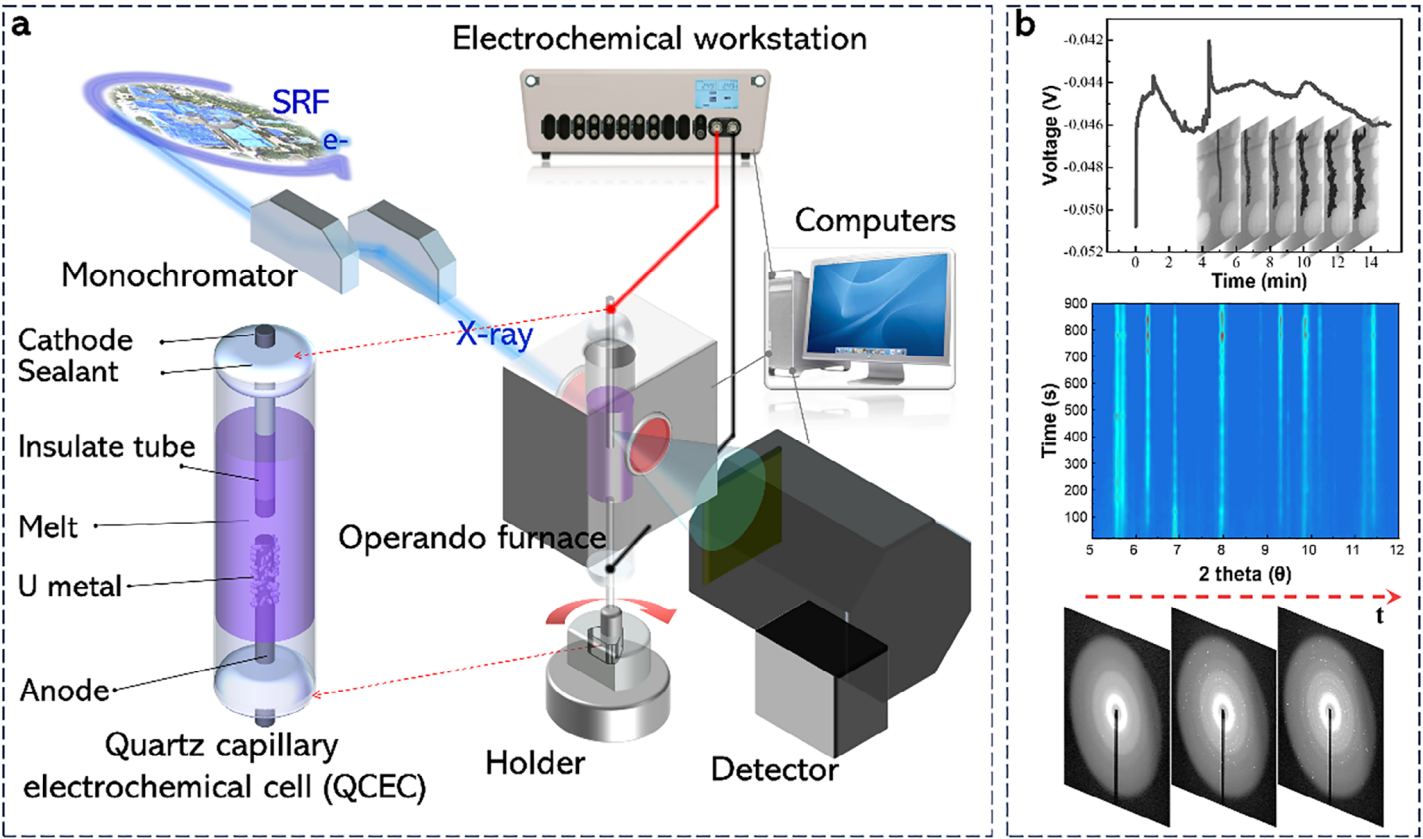
Operando SR‐µCT/HEXRD experimental setup to characterize uranium dendrite growth in LiCl‐KCl‐(4.78 wt%) UCl_3_ melt at 673 K. a) Schematic of the SR‐µCT/HEXRD experimental setup coupling with the self‐designed high temperature QCEC. b) The representative voltage transit of galvanostatic electrolysis and the typical X‐ray projection images and diffraction images extracted from SR‐µCT/HEXRD analysis.

The evolution of uranium dendrite growth in the electrochemical deposition of LiCl‐KCl‐UCl_3_ molten salt at 673 K was visualized by the operando SR‐µCT experiment performed at the 4W1A beamline at Beijing Synchrotron Radiation Facility (BSRF). Following each SR‐µCT, the operando HEXRD analysis was conducted to probe the microstructure evolution of the electrodeposited uranium by using the BL13SSW beamline of the Shanghai Synchrotron Radiation Facility (SSRF). **Figure** **1**a shows the schematic of the SR‐µCT/HEXRD setup coupled with the high‐temperature QCEC for the experiment. In the QCEC, the main body was fabricated using a three‐layer capillary quartz tube with a stainless steel (STS) working electrode (≈50 µm in diameter) and a uranium pre‐plated nickel counter electrode (≈100 µm in diameter) equipped at each end. To avoid the exposure to oxygen and moisture in the atmosphere, both ends of the QCEC were sealed by high temperature sealants after the salt loading inside a glovebox filled with inert argon gas. The electrodeposition of uranium was achieved through the galvanostatic electrolysis (Figure 1b) conducted using an electrochemical workstation as shown in Figure 1a. During the electrodeposition process, both SR‐µCT and HEXRD were employed continuously to collect X‐ray imaging and diffraction data on the grown uranium at STS working electrode. The representative X‐ray projection images and diffraction images extracted from SR‐µCT/HEXRD analysis are shown in Figure 1b.

### 2.2 Visualization and Quantification of Uranium Dendrite Growth and Ion Diffusion in Molten Salt

**Figure 2:**
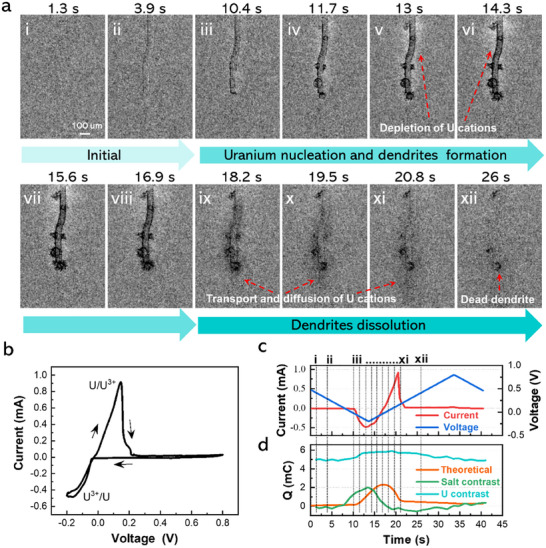
Operando 2D SXR to characterize the uranium redox reaction in CV scan. a) The processed δC for the evolutions of uranium electrodeposition and dissolution at different time during one CV full scan. The acquisition time is 1s for each frame. b) The recorded CV of LiCl‐KCl‐(4.78 wt%) UCl_3_ melted at 673 K with a scanning rate of 50 mV s^−1^ for operando 2D SXR test in (a). c) The variations of current and voltage with time for the CV scan in (b). d) The calculated electric charge Q variations of uranium based on the image contrasts of the electrodeposited uranium and diffusion layer in the salt, along with the theoretical electric charge variations in the CV scan.

In the operando SR‐µCT test, a 2D synchrotron X‐ray radiography (SXR) performed first in the QCEC can visually capture the uranium deposition and dissolution at the STS working electrode as well as the mass transfer of uranium at the interface of electrode/molten salt during the cyclic voltammetry (CV) scan of UCl_3_ (4.78 wt%) in LiCl‐KCl eutectic molten salt with a superior temporal and spatial resolution (see Movie S1, Supporting Information). The typical X‐ray projection images captured on the STS working electrode at different times in the CV scan are displayed in Figure S1 (Supporting Information). To enhance the image contrast of the electrodeposited uranium on the electrode and better identify the evolution process of uranium redox reaction in molten salt, the original X‐ray projection images captured at different times were further processed as contrast difference (δC) images by subtracting the absorption intensity of the X‐ray projection image taken at the beginning of CV scan. **Figure** **2**a shows the processed δC images for the evolution of uranium electrodeposition and dissolution at different times during one CV full scan (Figure 2b). The current and voltage variations with time in the CV scan are shown in Figure 2c, in which the time points for capturing δC images are labeled as i–xii.

From Figure 2a, it can be clearly seen that the uranium is gradually electrodeposited from molten salt and grows in the form of a dendrite in the reduction process of the CV scan (iii–viii: 10.4–16.9 s). Furthermore, the diffusion layer of uranium cation in the salt exhibiting as the bright area at the salt/electrode interface, displayed in Figure 2a can also be observed through the 2D SXR in this study. The diffusion layer is found to expand continuously along with the reduction of uranium cation in the CV scan (iii‐viii: 10.4–16.9 s). On the other hand, during the oxidation reaction process in the CV scan (x–xii: 18.2–26 s), the grown uranium dendrite dissolves, and the diffusion layer disappears slowly with the feeding of uranium cation from the oxidation of the electrodeposited uranium metal at the electrode. A few dead uranium dendrites are still left after one full cycle of CV scan, as displayed in Figure 2a–xii, which might be due to the irreversibility of the electrochemical reaction of UCl_3_ in LiCl‐KCl eutectic molten salt. To the best of our knowledge, this is the first time that the in situ observation of the variations of the diffusion layer and uranium dendrite visually in a CV scan.

Based on the image contrasts of the electrodeposited uranium and the diffusion layer shown in Figure 2a, the transferred electric charge (Q) of uranium can be calculated. Figure 2d shows the variations of the calculated electric charge of uranium during the CV scan in Figure 2b. The calculated electric charge of uranium increases with the electrodeposition of uranium in the reduction process of the CV scan, while it decreases in the oxidation process because of the uranium dissolution. In Figure 2d, the magnitude of the calculated electric charge of uranium based on the image contrast of the diffusion layer in the salt is close to that of the theoretical electric charge. However, there is a discrepancy with respect to the time point at which the electric charge begins to increase. This is within expectation since the electrocrystallisation occurring at the electrode surface could lag the diffusion of uranium cations in the salt melt. Compared with the theoretical electric charge of uranium as shown in Figure 2d, the magnitude of the calculated electric charge based on the image contrast of the electrodeposited uranium is much higher. This might be due to large uncertainties existing in the calculation brought by the tiny amount of the electrodeposited uranium in the CV scan (the size of the electrodeposited uranium is much less than that of the salt melt region).

**Figure 3:**
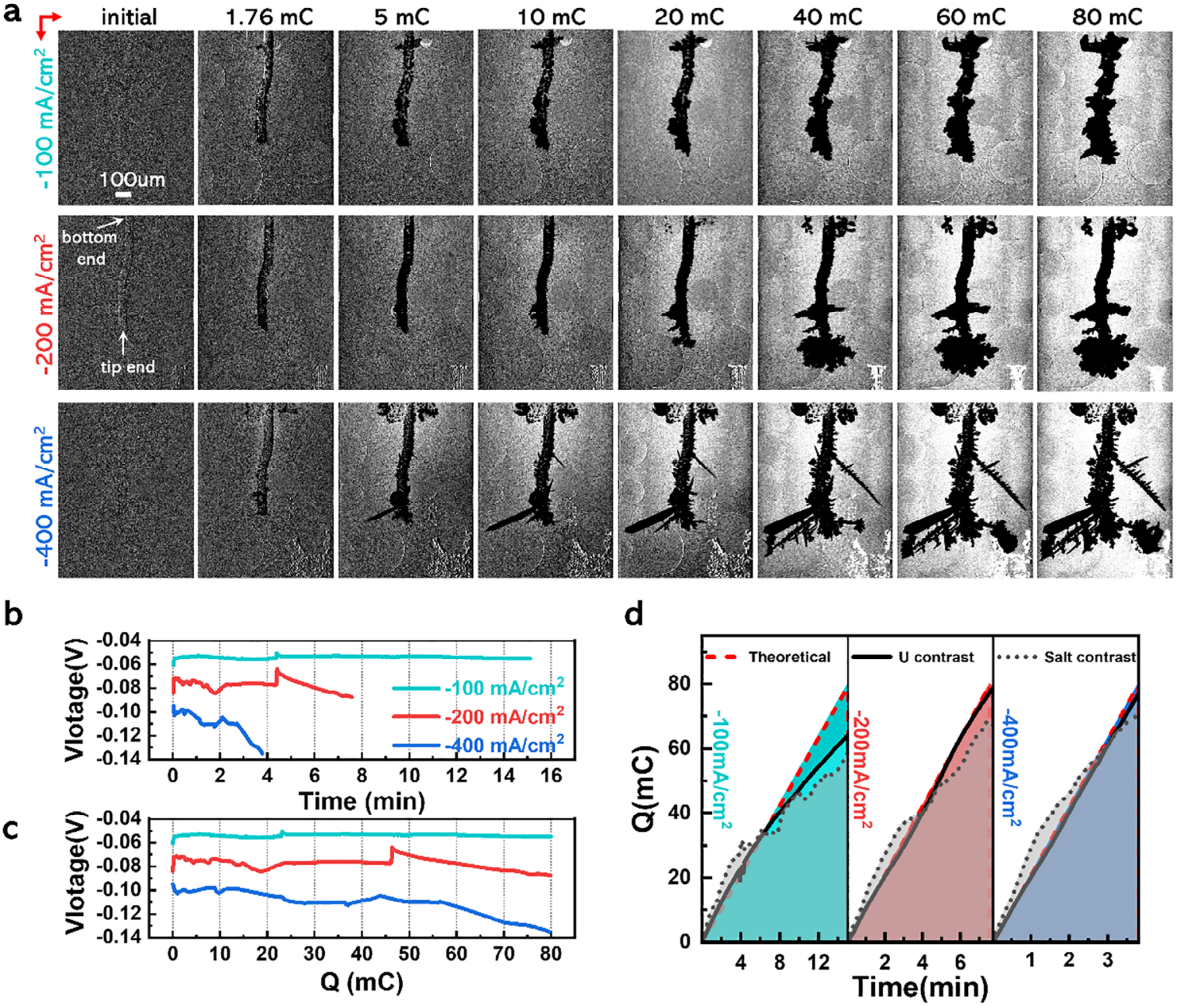
Operando 2D SXR analysis for the effect of electrolysis current density on uranium dendrite growth. a) The representative δC images for the evolutions of uranium electrodeposition in the electrolysis performed at different current densities. The balls in the images are bubbles adhering to the inner wall of the capillary. b) The recorded voltage variations along with time during the electrolysis. c) The recorded voltage variations along with the electric charge transferred during the electrolysis. d) The calculated electric charge variations of uranium with time based on the image contrasts of the electrodeposited uranium and diffusion layer in the salt, along with the theoretical electric charge.

To further study the evolution of uranium dendrite growth, the galvanostatic electrolysis was performed in LiCl‐KCl‐(4.78 wt%) UCl_3_ melt at 673 K with different current densities and the 2D SXR was employed continuously to collect the X‐ray projection images. **Figure** **3**a and Movie S2 (Supporting Information) show the processed δC images for the morphology evolution of uranium dendrite growth when different current densities (−100, −200, and −400 mA cm^−2^) are applied in the electrolysis. The corresponding original X‐ray projection images are shown in Figure S2 and Movie S2 (Supporting Information).

From Figure 3a, the applied electrolysis current density is found to play a critical role in the morphology of the grown uranium dendrite. Comparing the δC images taken when the same amount of electric charge is transferred under different electrolysis current densities, the electrodeposited uranium at a lower electrolysis current density is more compact and even. At the electrolysis current density of −200 mA cm^−2^, the uranium grows in the shape of a flaky, fan‐shaped dendrite at the tip end of the working electrode. While at the electrolysis current density of −400 mA cm^−2^, the uranium dendrite develops in a blade‐like structure, and the secondary branches start to appear and spread until hitting the inner wall of the capillary.

When looking into the bright area representing the diffusion layer of uranium cation at the electrode/molten salt interface, the bright area at the electrolysis current density of −100 mA cm^−2^ is evenly distributed around the electrode, as shown in Figure 3a. The thickness of this bright area almost remains the same after the electrolysis is performed for a certain time (after 20 mC), implying the timely supplementation of uranium cations from the bulk salt. This is consistent with the stable voltage variation as a function of time and electric charge transferred in the electrolysis process at −100 mA cm^−2^ (Figure 3b,c). The bright area becomes thicker and exhibits a higher brightness at the electrolysis current densities of −200 and −400 mA cm^−2^. It indicates that the diffusion layer of uranium cation thickens and the concentration of uranium cation in the diffusion layer decreases. In addition, the thickness of the bright area is found to expand as the electrolysis proceeds under the current densities of −200 and −400 mA cm^−2^, implying the inadequate replenishment of uranium cation limited by diffusion. The uranium depletion in the expanded bright area (diffusion layer) could bring an increase of overpotential, resulting in the negative shift of the voltage with time/electric charge in the electrolysis process, as displayed in Figure 3b,c.

The transferred electric charge of uranium was calculated based on the image contrasts of the electrodeposited uranium and the diffusion layer shown in Figure 3a. Figure 3d shows the calculated electric charge and the theoretical one for uranium during the galvanostatic electrolysis. Unlike what's found in the CV process, as shown in Figure 2d, the calculated electric charge based on the image contrast of the electrodeposited uranium agrees well with the theoretical electric charge, especially at high electrolysis current density. On the contrary, there is a large discrepancy between the theoretical electric charge and the calculated one from the image contrast of the diffusion layer in salt melt. The diffusion layer out of the imaging window or covered by the large amount of the electrodeposited uranium, resulted from galvanostatic electrolysis, was not counted in the image contrast analysis, which might be the main reason for this conflict phenomenon.

**Figure 4:**
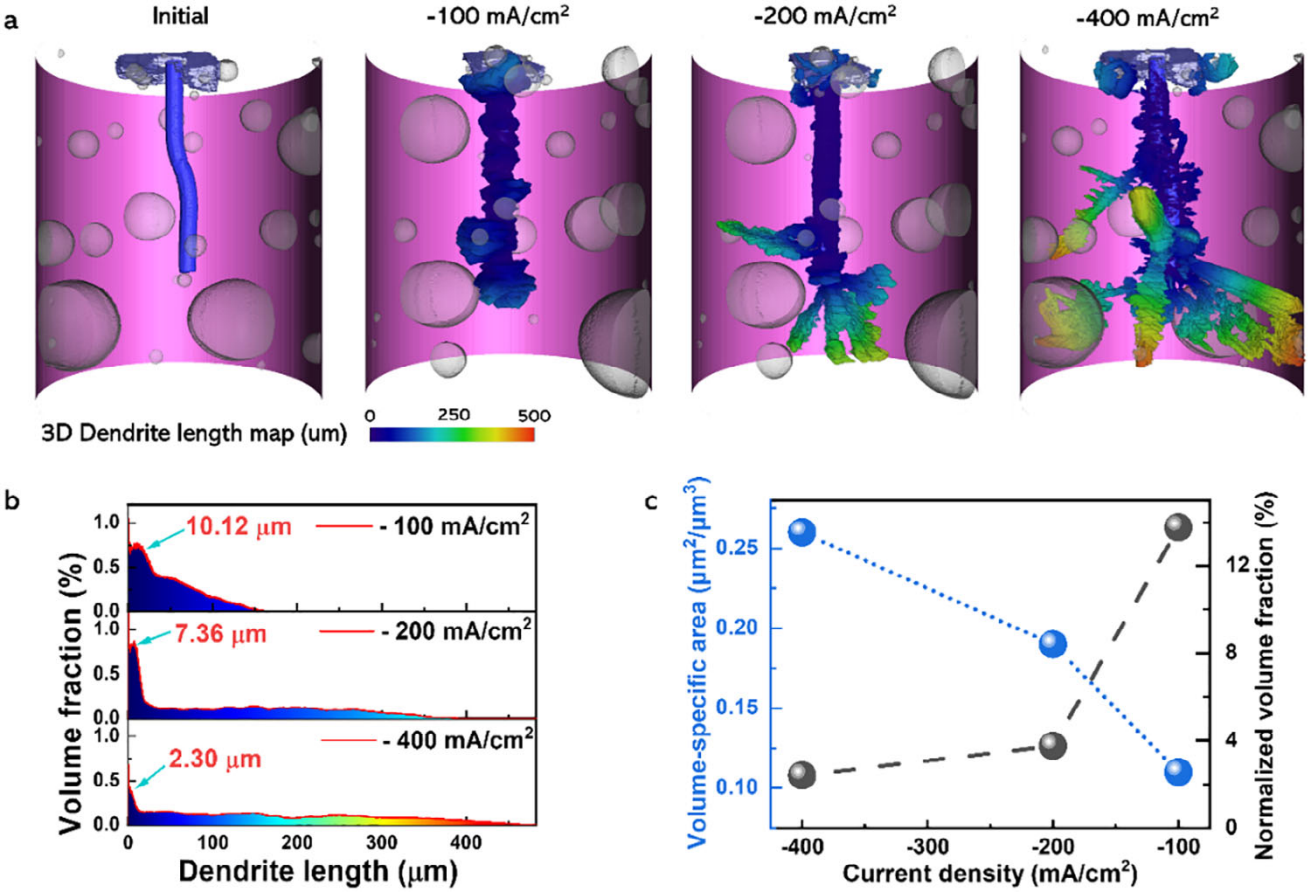
The 3D reconstruction and quantification analysis of the electrodeposited uranium dendrites under different electrolysis current densities. a) The reconstructed 3D images of the working electrode and the electrodeposited uranium dendrites. b) The volume distribution of the electrodeposited uranium dendrite, with its length calculated based on the reconstructed 3D images. c) The variations of the volume‐specific area and normalized volume fraction with electrolysis current densities.

To obtain more detailed information on the geometrical structures and dimensions of the electrodeposited uranium dendrites, the SR‐µCT results—reconstructed from a series of 2D SXR measured at different angles (Movie S3, Supporting Information), were further processed to create a 3D structure. The resulted 3D reconstruction images for the uranium dendrites formed when 80 mC of electric charge is transferred under different current densities are shown in **Figure** **4**a and Movie S3 (Supporting Information). Moreover, the reconstructed masses are very close to that of the theoretical ones (see Table S1, Supporting Information). It is found the electrodeposited uranium presents polyhedral shapes with sharp edges and corners (Figure S3, Supporting Information) when the uranium is electrolyzed at −100 mA cm^−2^. While at the electrolysis current density of −200 mA cm^−2^, flaky fan‐like (or moss‐like) structures appear at the tip end of the electrode although the rest part is mainly covered by uniform dense uranium film (Figure S3, Supporting Information). The morphology of the electrodeposited uranium under the electrolysis current density of −400 mA cm^−2^ is diverse, varying from tiny (<50 µm in length) flake‐like to large (≈500 µm in length) blade‐like dendrite structures.

The volume distribution of the electrodeposited uranium dendrite as a function of its length from the cathode surface was quantified using the reconstructed 3D images, with the results presented in Figure 4b. This analysis reveals the differential volume of metallic uranium present within successive length intervals along the dendrites. For electrolysis current densities of −100, −200, and −400 mA cm^−^^2^, the volume fraction exhibits distinct maxima at dendrite lengths of ≈10.12, 7.36, and 2.30 µm, respectively. These peaks correspond to the lengths at which the differential volume contribution of uranium metal is greatest. In the region extending from the cathode surface (length = 0) up to these respective peaks, the accumulated uranium volume increases monotonically. This trend is consistent with the initial nucleation and subsequent growth or agglomeration of uranium deposits covering the cathode surface, as visualized in Figure 3 and Movie S2 (Supporting Information), leading to an increasing material volume density close to the substrate. Conversely, beyond these peak lengths, particularly evident at the higher current densities (−200 and −400 mA cm^−^^2^), the uranium volume per unit dendrite length decreases sharply, eventually reaching a relatively stable, low value. This significant reduction suggests a transition toward a more anisotropic growth regime, characterized by dendrite elongation away from the cathode with less significant radial thickening at greater distances. The final plateau region implies that sustained growth to extended lengths is predominantly confined to a smaller population of dendrites. The volume and surface area of the electrodeposited uranium were also extracted (see Table S1, Supporting Information) based on the 3D‐reconstructed images in Figure 4a. The volume‐specific area, defined as the ratio of the surface area to volume for evaluating the compactness of the electrodeposited uranium dendrites, was calculated and displayed in Figure 4c. The volume‐specific area was found to increase with the increasing electrolysis current density, indicating that the higher current density can result in a lower compactness of uranium dendrites. The normalized volume ratio used to evaluate the space utilization of the electrodeposited uranium, as defined in Figure S4 (Supporting Information), was calculated as well and shown in Figure 4c. The higher normalized volume ratio at lower electrolysis current density demonstrates that the high space utilization and denseness of the electrodeposited uranium can be obtained by lowering the electrolysis current density applied.

### 2.3 Multiphysics Field Simulation of Electrolytic Process

**Figure 5:**
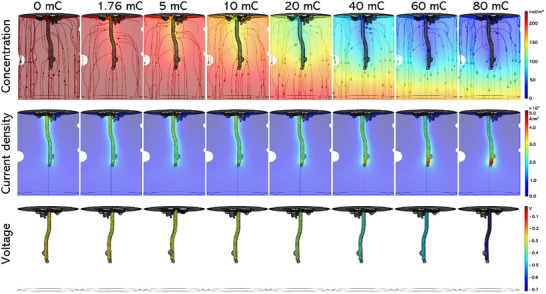
The variations of the uranium cation concentrations, current density, and voltage with the electric charge transferred during the electrolysis process at −400 mA cm^−2^ by Multiphysics field simulation.

To elucidate the mechanism of uranium dendrite growth and evolution, the 3D reconstructed geometry of the capillary cell in the viewport (Figure S5, Supporting Information) was taken to perform the multiphysics simulation. **Figure** **5** shows the variations of the simulated concentration, current density, and electric field with the electric charge transferred (also electrolysis time) during the electrolysis process at the current density of −400 mA cm^−2^ (results for the electrolysis current densities of −100 and −200 mA cm^−2^ are displayed in Figures S6–S8, Supporting Information). It is found that the uranium concentration in the bulk salt is axially symmetric in the radial space of the electrode throughout the whole electrolysis process, although the concentration streamline distribution varies due to the limited geometry and gas bubbles stuck to the inner wall of the capillary. As the electrolysis proceeds, the uranium cations in the salt around the electrode deplete, and the depletion area in the radial space expands gradually. There is also a concentration gradient in the axial space with higher concentration at the tip end of the working electrode. This is expected since the tip end of the working electrode is closer to the anode. The uranium cation around the tip end of the working electrode is easier to be easily replenished from the uranium anode. The change of the simulated concentration with electric charge transferred at different electrolysis current densities is similar to the image contrast variations observed in Figure 3a. This validated the multiphysics simulation model developed in this study.

The current density in Figure 5 shows its distribution along the electrode remains almost the same with time before the electric charge of 20 mC is transferred. In addition, the high current density zone is found to be located at the bottom end of the electrode. However, the current density at the tip end of the electrode increases dramatically with electric charge when the charge transferred is above 20 mC. Consequently, the high current density zone moves from the bottom end of the electrode toward the tip end. It is noteworthy that the current density is mainly distributed at the electrode surface, and the current density in the salt melt is steady at a relatively low value. Considering that the variation of current density mainly results from the migration transition of uranium cations, the migration and diffusion of uranium cations in the salt melt should be relatively stable. The simulated voltage in Figure 5 shows that its distribution along the electrode is homogenous. As the electrolysis proceeds, the voltage of the electrode shifts negatively with the electric charge transferred. Comparing the voltage variations under different electrolysis current densities, as shown in Figure S8 (Supporting Information), the voltage fluctuations of the electrode at lower current densities are very limited due to the timely replenishment of uranium cations from the anode during the electrolysis process. The insufficient replenishment of uranium caused by the rapid depletion at high electrolysis current density leads to the drastic fluctuations in the actual current density and voltage at the electrode surface, which is the critical factor in the kinetically anisotropic growth of uranium dendrites. This also explained the larger uranium dendrites grown at high electrolysis current density.

### 2.4 Crystal Structure and Orientation Evolution of Uranium Dendrite Growth

**Figure 6:**
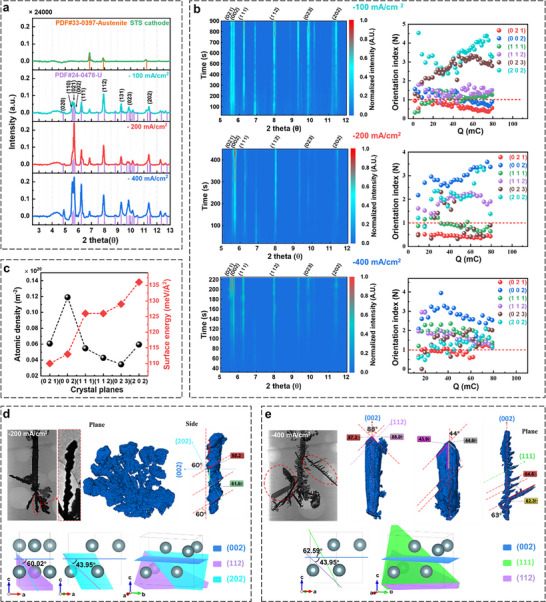
Operando HEXRD of uranium dendrite growth under the electrolysis current densities of −100, −200, and −400 mA cm^−2^. a) XRD patterns of the STS working electrode and the electrodeposited product when 80 mC of electric charge is transferred in the electrolysis performed under different current densities. b) The variations of the XRD pattern and dominant planes during the electrolysis. c) The calculated surface energy and atomic density for different planes of uranium. d,e) Growth pattern and angle analysis of the Uranium dendrites obtained at (d) −200 and (e) −400 mA cm^−2^ based on 3D reconstruction and the orthorhombic cell of α‐U, respectively.

Operando HEXRD was also performed to in situ study the evolutions of the crystal structure and orientation of the grown uranium dendrite during the electrolysis. **Figure** **6**a shows the obtained XRD patterns of the STS working electrode and the electrodeposited product when 80 mC of electric charge is transferred in the electrolysis performed under different current densities. The diffraction peak of the working electrode is identified as the austenite crystal phase. The displayed XRD pattern of the electrodeposited product in Figure 6a is the one with its background (molten salt, capillary, and electrode) subtracted, and the metallic α‐uranium phase is identified from the diffraction peaks.

The XRD patterns of the electrodeposited uranium were acquired continuously during the electrolysis performed under different current densities, and the diffraction peak intensity variations with electrolysis time were plotted as color maps shown in Figure 6b. From Figure 6b, it is clear that the peak intensities of the (021), (002), (111), (112), (023), and (202) planes in metallic α‐uranium increase with the electrolysis time, indicating the accumulation of the electrodeposited uranium. The relative ratio of each plane is defined as the orientation index (*N*) (see “Experimental Section” for further details), and their variations during electrolysis were extracted based on the obtained XRD patterns. The variations of the relative ratios of the six main planes of α‐uranium with electric charge displayed in Figure 6b show that (202) and (023) planes are dominant in the electrodeposited uranium through the whole electrolysis process at the current density of −100 mA cm^−2^. While under the current densities of −200 and −400 mA cm^−2^, the dominant planes are (002), (202), (112) and (002), (111), (112), respectively. According to the inter‐plane spacing order of (002) > (111) > (112) > (023) > (202),^[^ ^12^ ^]^ the ratio of the preferentially oriented planes with larger inter‐plane spacing increases with the electrolysis current density. This might explain the loose and large‐sized uranium dendrites grown at high electrolysis current density as observed from the SR‐µCT.

The surface energy and atomic density of different crystal planes are also calculated and shown in Figure 6c. It is found that the dominant planes of (202) and (023) in the uranium electrodeposited under the electrolysis current density of −100 mA cm^−2^ have high surface energy while relatively low atomic density. On the contrary, the most dominant plane (002) in the uranium formed under the electrolysis current densities of −200 and −400 mA cm^−2^ has low surface energy and high atomic density. In general, the system with high energy has the tendency to move toward the low‐energy state. The high energy of the dominant planes of the uranium formed under the current density of −100 mA cm^−2^ will preferentially move toward the low energy state by reducing its surface area with a strong adsorption force. Thus, the uranium electrolyzed at −100 mA cm^−2^ should be more compressible than the grown uranium under the electrolysis current densities of −200 and −400 mA cm^−2^. Moreover, the higher the atomic density of a crystal plane, the easier it is for atoms to stack on. The limited diffusion and restricted replenishment of uranium cations will only favor the growth of crystal planes where the atoms are inclined to stack, for instance, the (002) plane. The preferential growth of the (002) crystal plane leads to the formation of large‐sized dendrites.

The uranium dendrites captured by SR‐µCT in the X‐ray projection images can also be reconstructed for the dendrite growth angle analysis. Figure 6d shows the analysis results of the dendrite pieces taken from the electrodeposited uranium under the electrolysis current densities of −200 and −400 mA cm^−2^. The electrodeposited uranium under the electrolysis current density of −100 mA cm^−2^ was not processed due to the small size of the grown dendrite. From Figure 6d, the angle between the plane and the arrangement direction of the crystal nuclei in the cross section of the typical flaky uranium dendrite piece formed under the electrolysis current density of −200 mA cm^−2^ is ≈60°, which is close to the angle of 60.02° between (002) and (202) planes. Therefore, the electrodeposited uranium at the electrolysis current density of −200 mA cm^−2^ is speculated to grow and accumulate on the (202) and (112) crystal planes, and to exhibit lateral expansion along the (002) plane. While at −400 mA cm^−2^, the angle between the central axis and oblique axis at the tip of the blade‐shaped dendrite is ≈44°, which is close to the angle of 43.95° between (002) and (112) planes. In addition, the branching angle of ≈63° of uranium branches is close to the angle of 62.6° between (002) and (111) planes. It means that blade‐shaped uranium dendrite grows along the (002) plane direction and stacks up on the (112) crystal plane, while the small branches grow along the (002) plane direction but stack up on the (111) crystal plane.

## 3 Discussion

The present study investigated the evolution of uranium dendrite growth in HTMS electrochemistry through the developed operando characterization method based on SR‐µCT and HEXRD. The morphologies of the grown uranium dendrites and ion diffusion layer exhibit a high discrepancy when different current densities were applied in the galvanostatic electrolysis. A stable diffusion layer can be formed with the proceeding of electrolysis at low current density, which results in a compact and even uranium electrodeposition. However, the expanding ion diffusion layers and anisotropic growth of uranium dendrites were observed during the electrolysis under the high current densities of −200 and −400 mA cm^−^^2^. The electrochemical reaction kinetics at the electrode could be faster than the ion diffusion when a high electrolysis current density is applied, and the electrochemical reaction is limited or controlled by the ion diffusion process. The expanding diffusion layer observed at high electrolysis current density indicates that the depleted uranium cation cannot be timely replenished, and the reaction is controlled by the ion diffusion process. This is also reflected in the drastic variations of the current density and voltage monitored at the electrode surface during the electrolysis process. Therefore, the limited diffusion resulting from the slow transport of ions or high reaction rate occurring at the electrode should be the main reason for the formation of uranium dendrites in HTMS electrochemistry. Although a similar conclusion was given in our previous studies,^[^ ^13^ ^]^ this work represents the first time elucidating the dendrite formation principle through the in situ visualization of the whole growth process of uranium dendrite in molten salt under these specific micro‐capillary conditions.

Unlike the critical factor of limited ion diffusion resulting in the formation of uranium dendrites, the growth morphology of uranium dendrites appears to be strongly influenced by its crystal structure and orientation. The in‐situ HEXRD analysis provided insights into the crystal structure during electrolysis. It is important to note, however, that these in‐situ crystallographic data were obtained under specific micro‐capillary conditions in a single experimental run and thus represent observations whose repeatability awaits confirmation. The significant differences in scale, mass transport, and electric field distribution between this micro‐environment and macroscale systems must also be considered when interpreting these findings. Within this context, the analysis suggested that the dominant crystallographic orientations may differ when different electrolysis current densities are applied. Under the conditions studied, planes with larger inter‐planar spacing appeared to be more prevalent at higher current densities. This observation seems to correlate with the looser and large‐size uranium dendrites grown at −200 and −400 mA cm^−^^2^ as visualized by SR‐µCT, suggesting a potential link between crystallographic texture and macroscopic morphology in these early growth stages captured in situ. Furthermore, the observed angles between branches and trunks in the uranium dendrites appeared consistent with characteristic angles related to the uranium crystal structure, hinting that the crystallographic orientation likely influences the specific dendritic shape. These observations suggest that manipulating the preferred crystallographic orientation, if possible, could potentially offer a strategy to alter dendrite shape and suppress growth, although further investigation under varying conditions and validation of repeatability are required to firmly establish this relationship.

Overall, while limited ion diffusion appears to be a critical factor for the initiation of uranium dendrites in HTMS, these in‐situ observations suggest that crystal plane orientation plays a significant role in guiding the subsequent growth morphology and evolution under the specific micro‐electrochemical conditions studied. These findings provide significant insights into the uranium dendrite growth mechanism under these conditions and offer potential directions for the further development of dendrite suppression strategies. Although uranium is focused on in this study, the discoveries and operando characterization method developed could be applied in a wide range of applications in other fields, such as fuel cells, batteries, metallurgy, etc.

## 4 Experimental Section

### Salt Preparations

Lithium chloride‐potassium chloride eutectic (51:49 mol%, >99.9%) was purchased from Alfa Aesar. To remove as much water and oxygen in the salt as possible, the salt was placed in a glassy carbon crucible in a furnace in an argon atmosphere glove box (99.99% Ar, H_2_O < 0.5 ppm, O_2_ < 0.1 ppm) and melted at 773 K, then a stream of Ar (10% HCl) gas was introduced into the melt for at least 2 h. For the preparation of the uranium‐containing salt, BiCl_3_ (>99.9%, Merklin) and uranium metal (≈40 g) were used to introduce UCl_3_ into the LiCl‐KCl eutectic melt.^[^ ^14^ ^]^ The concentration of U was determined by the inductively coupled plasma optical emission spectrometry (ICP‐OES, Agilent 5110). In this work, all the experiments were fixed and performed in a LiCl‐KCl‐4.78 wt% UCl_3_ melt.

### Quartz Capillary Electrochemical Cell

The quartz capillary electrochemical cell (QCEC) is fabricated using three‐layer quartz capillary tubes (Kejing glass). The innermost quartz capillary tube (≈100 µm in diameter) acts as an insulating sheath for the STS cathode (50 µm). The intermediate quartz tube (inner diameter: 0.8 mm, length: 90 mm) was a reaction tube containing salt and two electrodes. To eliminate mechanical vibration of the cell during the collection of 3D data acquisition and to optimize practical operability, an outer quartz tube of 0.08 mm thickness was chosen as a protective tube. Both ends of the QCEC were sealed by the high‐temperature resistant sealant (Torr Seal, Agilent). A Ni wire (100 µm, 99.5%, Alfa Aesar) coated with a dense layer of uranium (15–20 mm long) deposited by pulsed current techniques acted as the anode. Electrolysis was carried out for 4000 s in a corundum crucible containing 40 g of LiCl‐KCl‐4.78 wt% UCl_3_ melt with a charge of 10 C. A copper wire (50 µm in diameter) was connected to each end of the cathode and anode as a junction for electrochemical experiments. The working electrode was a stainless‐steel wire (50 µm, type 304, >99.9%) with a quartz tube sheath (0.1–0.3 mm in diameter). The molten salt is drawn up to the inner capillary tube using a syringe and micromanipulator.

### Electrochemical Tests

An electrochemical workstation (Autolab PGSTAT101) was used to perform the electrochemical tests, including CV and GE at 673 K. The QCEC was mounted on a holder and placed in a programmable heating chamber (Figure S9, Supporting Information), which maintained the temperature with an accuracy of ±1 K.

### SR‐µCT

The operando SR‐µCT test was performed at the 4W1A station of the Beijing Synchrotron Radiation Facility (BSRF). The incident X‐ray energy is monochromatized at 20 keV. The X‐ray beam passed through the two Kapton windows of the heating chamber. The X‐ray projection images were collected by a qCMOS detector (Hamamatsu C15550, pixel array: 4096 × 2304) with an effective pixel size of 0.46 µm and a time resolution of 1 s. The photograph for the experimental setup is shown in Figure S9 (Supporting Information).

### HEXRD, Transmission Mode

The operando HEXRD experiment is performed at the beamline BL13SSW of the Shanghai Synchrotron Radiation Facility, which operates at 3.5 GeV electrons with a current of 210 mA. The incident X‐ray energy is monochromatized at 50 keV. A 2D detector is used to acquire X‐ray diffraction patterns in the transmission mode of the high‐energy and flux beamline with an effective pixel size of 139 µm and a time resolution of 2 s. The photograph for the experimental setup is shown in Figure S9 (Supporting Information).

### Visualization and Quantification

In the SR‐µCT experiments, the 2D projection X‐ray sequence in the CV tests was collected with an interval of 0.1 s and an exposure time of 1 s, while in the GE tests, it changes to a collection with an interval of 1 s and an exposure time of 4 s. As soon as the GE tests were completed, we immediately performed a 180° rotation acquisition (exposure time: 3 s) to collect the 3D data. The 2D X‐ray image represents the total X‐ray adsorption of the substance in the projection direction. By comparing the adsorption of the initial 2D image, the distribution map and total amount of uranium depositiontime‐dependent variation was obtained. The 3D data were reconstructed using Avizo software, and based on this, the material segmentation, quantitative data and 3D visualisation were achieved. Details of the data processing procedures and algorithm are described in Figure S10 (Supporting Information).

The mass of uranium could be calculated from the X‐ray adsorption of different materials. As shown in Figure S10 (Supporting Information), the transmission of the U, STS electrode, molten salt, quartz tube, and bubbles can be detected. The regions containing deposited uranium could be selected by the transmission threshold. The *Watershed Algorithm* in AVIZO was used for the species segmentation of 2D‐t and 3D images. For the 2D‐t data, the transmission can be expressed as:

(1) $$\begin{array}{c} T\;\left(x,y,t\right)=\exp\left(-\int \mu \left(x,y,z,t\right)dz\right)\;\\ =\exp\left(-\mu_{U}z_{U}\left(x,y,t\right)-\mu_{\mathrm{salt}}z_{\mathrm{salt}}\left(x,y,t\right)-\mu_{quartz}z_{quartz}\left(x,y,t\right)-\mu_{STS}z_{STS}\left(x,y,t\right)\right)\;\# \end{array}$$

where *µ*_U_, *µ*_salt_, *µ*_quartz_, and *µ*_STS_ are the adsorption coefficients of U, molten salt, quartz tube and STS electrode, respectively. z_U_(x,y,t) and z_salt_(x,y,t) are the corresponding penetration lengths of the X‐ray beam in the projection direction. From the values of T_0_ and T_1_, the deposited uranium can be calculated using the following equation:

(2) $$T_{0}\;\left(x,y,0\right)=\exp\left(-\mu_{salt}z_{salt}\left(x,y,0\right)-\mu_{quartz}z_{quartz}\left(x,y,0\right)-\mu_{STS}z_{STS}\left(x,y,0\right)\right)$$

(3) $$T_{1}\;\left(x,y,t\right)=\exp\left(-\mu_{U}z_{U}\left(x,y,t\right)-\mu_{salt}z_{salt}\left(x,y,t\right)-\mu_{quartz}z_{quartz}\left(x,y,t\right)-\mu_{STS}z_{STS}\left(x,y,t\right)\right)$$

In this equation, it is assumed that the thickness of the quartz tube and the STS electrode are constant across the X‐ray window, and that the change of z_U_ and z_salt_ is opposite. Therefore, the mass of uranium deposited at time t:

(4) $$m_{U}\;\left(t\right)=\rho_{U}\;\int \int -\frac{\ln\;(T_{1}\left(x,y,t)\right)-\ln\;(T_{0}\left(x,y,0)\right)}{\mu_{U}-\mu_{\mathrm{salt}}}\mathrm{dxdy}\;$$

The variation of uranium cations in the molten salt can also be calculated using the above equation. The image sequence of ln(T_1_/T_0_) is named as “δC‐t”, which can enhance the image contrast of the diffusion of U cations in molten salt.

For 3D data, the volume integral of the uranium dendrites must be weighted by the uranium volume fraction:

(5) $$m_{U}=\rho_{U}\;\int \int \int \;-\frac{\ln\left(T_{U}\left(x,\;y,\;z\right)\right)}{\mu_{U}-\mu_{salt}}dxdydz$$

as there is still some molten salt in the region defined as uranium (especially under high current conditions).

According to the 3D distribution of the uranium dendrites, we can obtain the distance from each pixel in the uranium dendrites to the STS electrode and obtain its histogram distribution. The specific operation is to use the *Geodesic distance* command in AVIZO to obtain the distance from each point in the 3D space to the STS electrode and then index the distance to each corresponding pixel in the uranium dendrite.

In HEXRD experiments, the incident X‐ray beam is partially absorbed, and the directly transmitted X‐ray is blocked by the beam stop. The diffracted X‐ray is detected on a 2D detector (pixel array: 3072 × 3072, pixel size: 139 µm) at a spacing of 200 mm. The angle between the diffracted and the incident X‐rays follows Bragg's law:

(6) 2dsinθ=nλ

Therefore, the integral of the diffraction ring with the same radius on the 2D detector is the corresponding diffraction intensity at angle 2θ.

The crystallographic orientation index (*N*) is a measure of the relative ratio of the preferentially formed facets of the U deposits and is given by^[^ ^15^ ^]^:

(7) $$N=\frac{\frac{I/I_{\left(hkl\right)}}{\sum I/I_{\left(hkl\right)}}}{\frac{JCPDS.I/I_{\left(hkl\right)}}{\sum JCPDS.I/I_{\left(hkl\right)}}}$$

where *I/I*_(hkl)_ is the ratio of diffraction intensities, *JCPDS. I/I*_(hkl)_ is the ratio of diffraction intensities of the JCPDS standard, and the sums represent the combined ratios of the total diffraction intensities for all the crystalline faces. A preferentially formed crystallographic orientation (facet) will have a value of N greater than 1.

### Modeling and Simulation

The modeling and simulation were performed by using COMSOL Multiphysics simulation. The physics model “tertiary current distribution and Nernst–Planck(tcd), supporting electrolyte” was used. The Nernst–Planck equation in the simulation is shown as follows:

(8) $$\;J_{i}=-D_{i}\nabla c_{i}-z_{i}u_{m,i}Fc_{i}\nabla \varphi_{i}$$

(9) $$\;u_{m,i}=\frac{D_{i}}{RT}$$

where *D*_i_ is the diffusion coefficient, *J*_i_ the reaction flux of the species, *c*_i_ the concentration of reactive cations, *F* the Faraday constant, *z*_i_ the valence, *u*_m,l_ the mobility, and φ_i_ the electrolyte potential.

The simulation electrolytic cell consists of electrolyte with a diameter of 0.8 mm, STS working electrode, U plating counter electrode and bubbles. The mesh reconstruction of the STS working electrode and bubbles was achieved by AVIZO. The geometry of the U plating anode was built by COMSOL. The currents of the anode and cathode are 88, 176, and 352 µA (corresponding to cathodic current densities of −100, −200, and −400 mA cm^−2^, respectively). The diffusion coefficient of U cations in the LiCl‐KCl molten salt was set to 1.5–7.0 × 10 ^−9^ m^2^ s^−1^. The initial concentration of UCl_3_ was 233.5 mol m^−3^.

The boundary conditions for the anode and cathode electrodes were given by the Butler–Volmer equation for U electrodeposition:

(10) $$\;i_{loc}=nFk_{0}\left(a_{U}exp\left(\frac{\alpha_{a}F\eta }{RT}\right)-a_{U^{3+}}exp\left(-\frac{\alpha_{c}F\eta }{RT}\right)\right)$$

The electrodeposition process was assumed to take place through the following reactions:

(11) $$\mathrm{Cathode}:\;U^{3+}+3\;e^{-}=U$$

(12) $$\mathrm{Anode}:\;U-3\;e^{-}=U^{3+}$$

### DFT Computational Method

All density functional theory (DFT) calculations in this work were performed by using the Vienna Ab‐Initio Simulation Package (VASP).^[^ ^16^ ^]^ The electron‐ion interaction was described by the projector augmented wave (PAW) method^[^ ^17^ ^]^ and the Perdew–Burke–Ernzerh of generalized gradient approximation (PBE‐GGA)^[^ ^18^ ^]^ exchange‐correlation functional was employed. In all calculations, the Gaussian electron smearing method with σ = 0.10 eV and the plane‐wave cutoff energy of 550 eV was used. The bulk structure of the uranium crystal with space group CMCM was optimized using the Monkhorst–Pack *k*‐point mesh^[^ ^19^ ^]^ (10 × 8 × 8). The lattice parameters and atomic positions were fully relaxed until the maximum force on each atom is less than 0.01 eVÅ^−1^. Then, based on the optimized bulk structure of the uranium crystal, all surface slab models were established with a vacuum thickness of 15 Å, and the atomic positions were fully relaxed. The surface energies of the slab models were calculated as

(13) $$\;E_{\mathrm{bulk}}=\frac{1}{2A}\;\times \left(E_{\mathrm{slab}}^{N}-N\times E_{u-\mathrm{atom}}\right)$$

where $E_{\mathrm{slab}}^{N}$ indicates the total energy of the slab containing *N* atoms of uranium, *E*_u − atom_ is the total energy of a bulk uranium crystal per atom.

## Conflict of Interest

The authors declare no conflict of interest.

## Author Contributions

K.L. and T.T. contributed equally to this work. K.L. performed conceptualization, investigation, methodology, visualization, validation, funding acquisition, data curation, software, formal analysis, wrote original draft, wrote reviewing and editing, sources, supervision, and project administration. T.T. performed investigation, data curation, software, formal analysis, visualization, and validation. Y.K.Z. investigation, wrote reviewing and editing, and data curation. Y.F.W. performed writing reviewing and editing. Z.M.B. performed investigation and data curation. S.F.W., K.Z., W.X.H., and J.R.Z. acquired sources and performed validation. W.Q.S. performed conceptualization, acquired sources, wrote reviewing and editing, and acquired funding acquisition.

## Supporting information



Supporting Information

Supplemental Movies

Supporting Information

## Data Availability

The data that support the findings of this study are available from corresponding author upon reasonable request.

## References

[1] a) K. Grjotheim , C. Krohn , M. Malinovsky , K. Matiasovsky , J. Thonstad , in Aluminum Electrolysis. The Chemistry of the Hall‐Heroult Process, Aluminium‐ Verlag GmbH, Dusseldorf, Germany 1977, p. 350;

[2] M. Mirza , R. Abdulaziz , W. C. Maskell , S. Wilcock , A. H. Jones , S. Woodall , A. Jackson , P. R. Shearing , D. J. Brett , Energy Environ. Sci. 2023, 16, 952.

[3] a) H. Yin , X. Mao , D. Tang , W. Xiao , L. Xing , H. Zhu , D. Wang , D. R. Sadoway , Energy Environ. Sci. 2013, 6, 1538;

[4] X. Lu , Z. Zhang , T. Hiraki , O. Takeda , H. Zhu , K. Matsubae , T. Nagasaka , Nature 2022, 606, 511. 35417651 10.1038/s41586-022-04748-4

[5] a) T. C. Totemeier , in Morphologies of Uranium Deposits Produced During Electrorefining of EBR‐II Spent Nuclear Fuel, Argonne National Laboratory, Argonne, IL (US) 2000, pp. 1–11;

[6] a) X. Lu , A. Bertei , D. P. Finegan , C. Tan , S. R. Daemi , J. S. Weaving , K. B. O'Regan , T. M. M. Heenan , G. Hinds , E. Kendrick , D. J. L. Brett , P. R. Shearing , Nat. Commun. 2020, 11, 2079; 32350275 10.1038/s41467-020-15811-x PMC7190643

[7] S. H. Yu , X. Huang , J. D. Brock , H. D. Abruna , J. Am. Chem. Soc. 2019, 141, 8441. 31062595 10.1021/jacs.8b13297

[8] a) B. Bozzini , C. Mele , A. Veneziano , N. Sodini , G. Lanzafame , A. Taurino , L. Mancini , ACS Appl Energy Mater. 2020, 3, 4931; 10.1021/acsaem.5c01110 PMC12308759 40747146

[9] X. Liu , A. Ronne , L. C. Yu , Y. Liu , M. Ge , C. H. Lin , B. Layne , P. Halstenberg , D. S. Maltsev , A. S. Ivanov , S. Antonelli , S. Dai , W. K. Lee , S. M. Mahurin , A. I. Frenkel , J. F. Wishart , X. Xiao , Y. K. Chen‐Wiegart , Nat. Commun. 2021, 12, 3441. 34108466 10.1038/s41467-021-23598-8 PMC8190292

[10] Y. Wang , A. P. Olson , C. Falconer , B. Kelleher , I. Mitchell , H. Zhang , K. Sridharan , J. W. Engle , A. Couet , Nat. Commun. 2024, 15, 3106. 38600068 10.1038/s41467-024-47259-8 PMC11271644

[11] a) H. B. Jiao , Z. L. Qu , S. Q. Jiao , Y. Gao , S. Shijie Li , W. L. Song , H. C. Chen , H. M. Zhu , R. Q. Zhu , D. N. Fang , Sci. Adv. 2022, 8, 1; 10.1126/sciadv.abm5678 PMC8827660 35138887

[12] C. Jacob , B. Warren , J. Am. Chem. Soc. 1937, 59, 2588.

[13] T. Tan , K. Liu , W. Shi , J. Nucl. Mater. 2024, 591, 154936.

[14] a) K. Liu , t. Tan , X. Zhou , N. Zheng , Y. Ma , M. Kang , B. Wang , Z. Chai , W. Shi , J. Nucl. Mater. 2021, 555, 153110;

[15] a) K. S. Willson , J. A. Rogers , Tech. Proc. Am. Electroplat. Soc. 1964, 51, 92;

[16] G. Kresse , J. Furthmüller , Phys. Rev. B 1996, 54, 11169. 10.1103/physrevb.54.11169 9984901

[17] P. E. Blöchl , Phys. Rev. B 1994, 50, 17953. 10.1103/physrevb.50.17953 9976227

[18] J. P. Perdew , K. Burke , M. Ernzerhof , Phys. Rev. L 1996, 77, 3865. 10.1103/PhysRevLett.77.3865 10062328

[19] H. J. Monkhorst , J. D. Pack , Phys. Rev. B 1976, 13, 5188.

