# Supplementary material for: Operando Characterization of Uranium Dendrite Growth in High‐Temperature Molten Salt Electrochemistry by Synchrotron X‐Ray Tomography and Diffraction
Source: Adv Sci (Weinh). 2025 Jun 11;12(32):e02345. doi: 10.1002/advs.202502345 (PMC12407277; doi:10.1002/advs.202502345)
Supplement: Supplementary file 1 — Supporting Information [file ADVS-12-e02345-s001.docx]

**Operando characterization of uranium dendrite growth in high temperature molten salt electrochemistry by synchrotron X-ray tomography and diffraction**

**Kui Liu^1,*,=^, Tan Tan^1,=^, Yuke Zhong^2,^ , Yafei Wang^3,^*, Tao Bo^4^, Zimei Bai^1^, Shanfeng Wang^2^, Kai Zhang^2,5^, Wanxia Huang^2^, Jianrong Zeng^6,7^, Weiqun Shi^2,3^***

^1^ Sino-French Institute of Nuclear Engineering and Technology, Sun Yat-sen University, Zhuhai, 519000, China

^2^ Institute of High Energy Physics, Chinese Academy of Sciences, Beijing 100049, China

^3^ School of Nuclear Science and Engineering, Shanghai Jiao Tong University, Shanghai 200240, China

^4^ Engineering Laboratory of Advanced Energy Materials Ningbo Institute of Materials Technology and Engineering, Chinese Academy of Sciences, Ningbo, 315201 China

^5^ University of Chinese Academy of Sciences, Beijing, 101407, China

^6^ Shanghai Synchrotron Radiation Facility, Shanghai Advanced Research Institute, Chinese Academy of Sciences, 201204 Shanghai, P. R. China.

^7^ Shanghai Institute of Applied Physics, Chinese Academy of Sciences, 201800 Shanghai, P. R. China.

^=^These authors contributed equally to this work.

*Corresponding authors:

Kui Liu: liuk75@mail.sysu.edu.cn

Yafei Wang: itsme@sjtu.edu.cn

Weiqun Shi: shiwq@sjtu.ac.cn


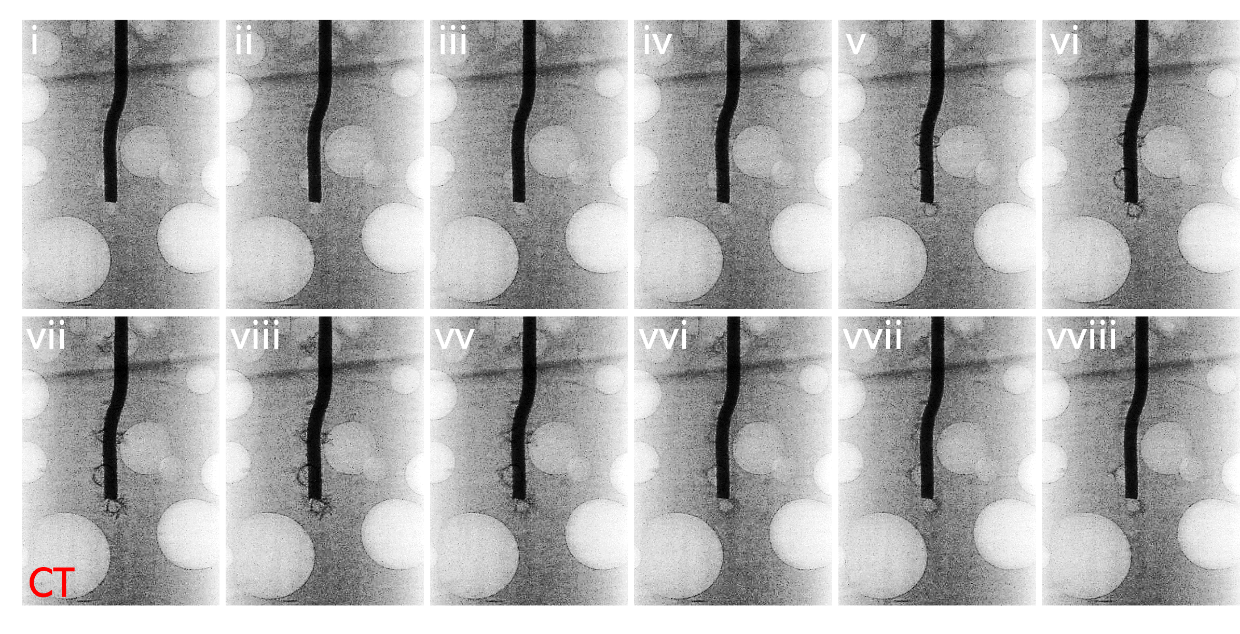


**Fig. S1.** Operando SR-μCT to characterize the uranium redox reactions of CV in LiCl-KCl-(4.78 wt%) UCl_3_ melt at 673K with a scanning rate of 50 mV/s. The representative X-ray projection images (as CT) with an acquisition time of 1s for each frame.


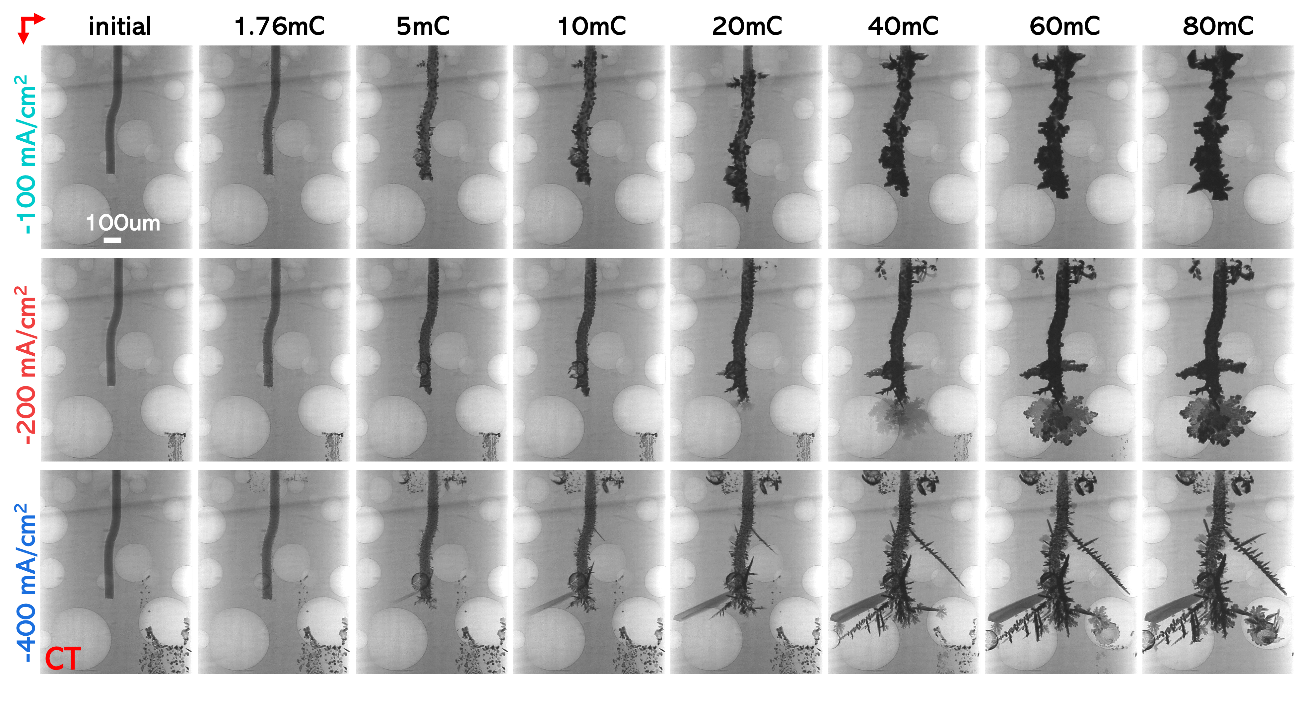


**Fig. S2.** Operando SX-µCT of uranium dendrites formation and effects of current density on morphology. Evolution of X-ray projection images of uranium dendrites formation at different current densities with an acquisition time of 5s for each frame.


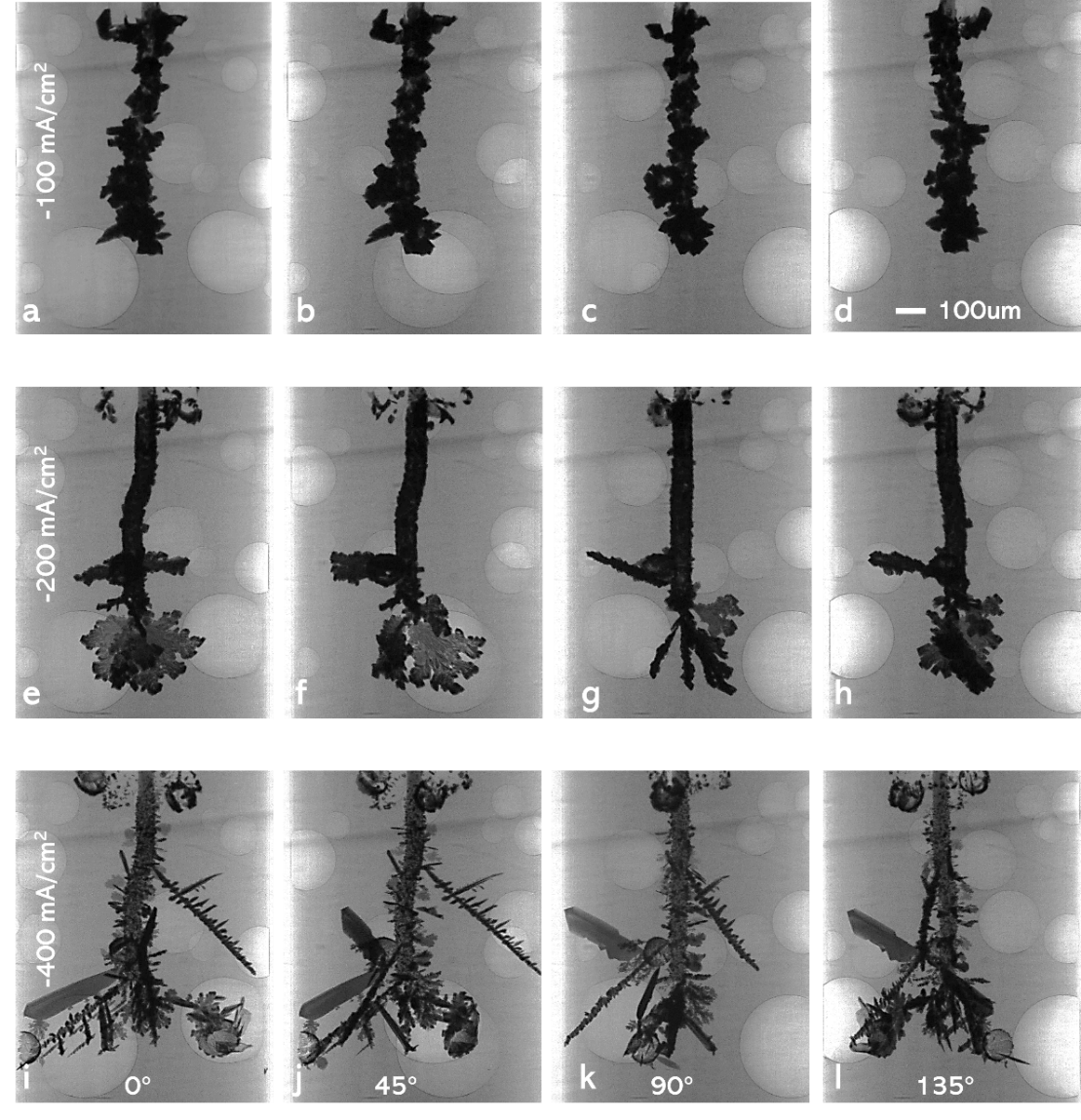


**Fig**. **S3.** Representative X-ray projection images of uranium dendrites viewed from different angels. The acquisition time is 4s for each frame.

**
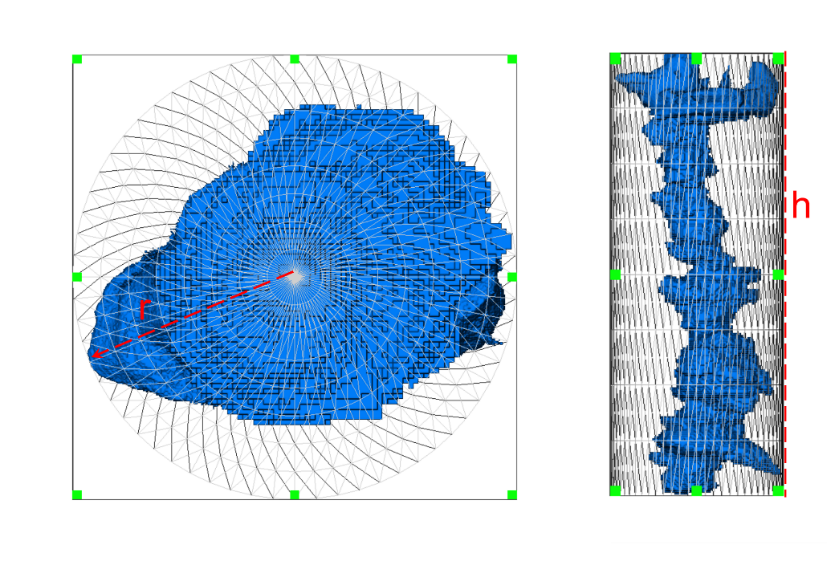
**

**Fig. S4**. The schematic for the definition of normalized volume ratio. Left: the top view of the uranium dendrite; right: the side view of the uranium dendrite. *h* and *r* represent the height and radius of the maximum uranium dendrite epitaxy. The percentage of uranium in the volume space (2πrh) is defined as the normalized volume fraction, and its magnitude allows a more pertinent evaluation of the space utilization of the electrolytic cell.


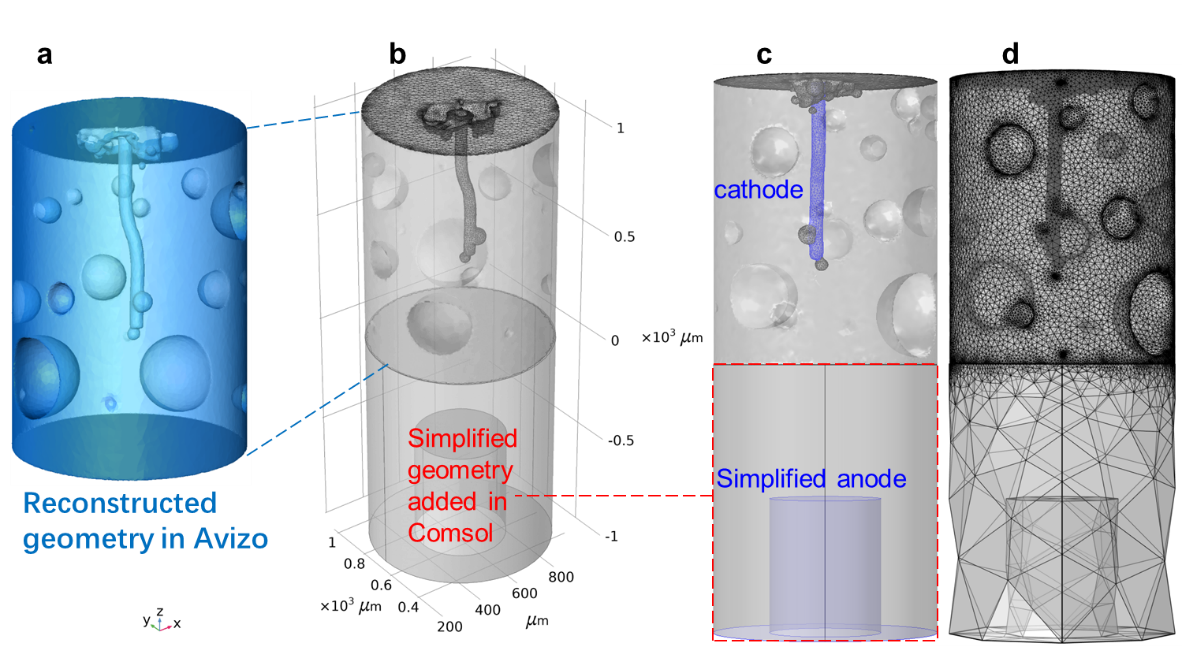


**Fig. S5**. (a) The reconstructed 3D geometry of the electrochemical test cell in the viewpoint achieved by AVIZO software; (b) the cell geometry built by COMSOL software with a simplified anodic region; (c) the geometries of cathode and anode; (d) the gridded electrochemical test cell.


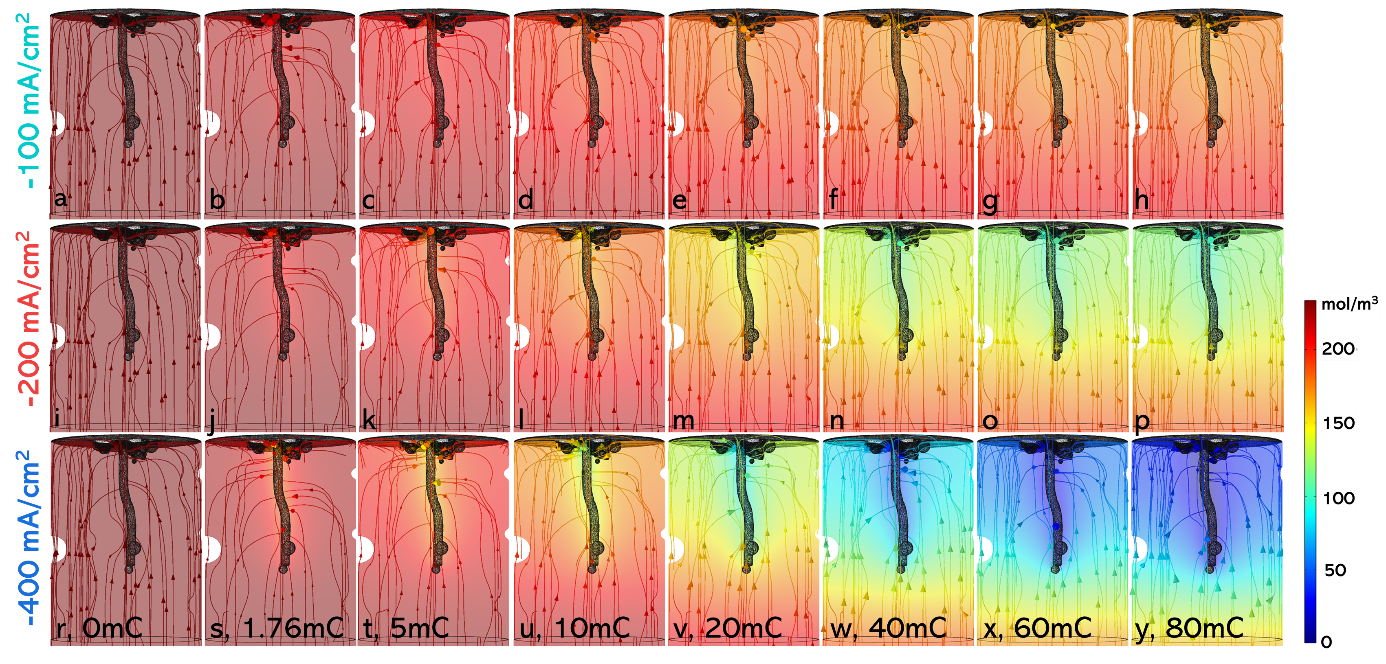


**Fig. S6**. The variations of the simulated uranium cation concentrations with the electric charge transferred during the electrolysis process at -100 mA/cm^2^, -200 mA/cm^2^, and -400 mA/cm^2^ by Multiphysics field simulation.


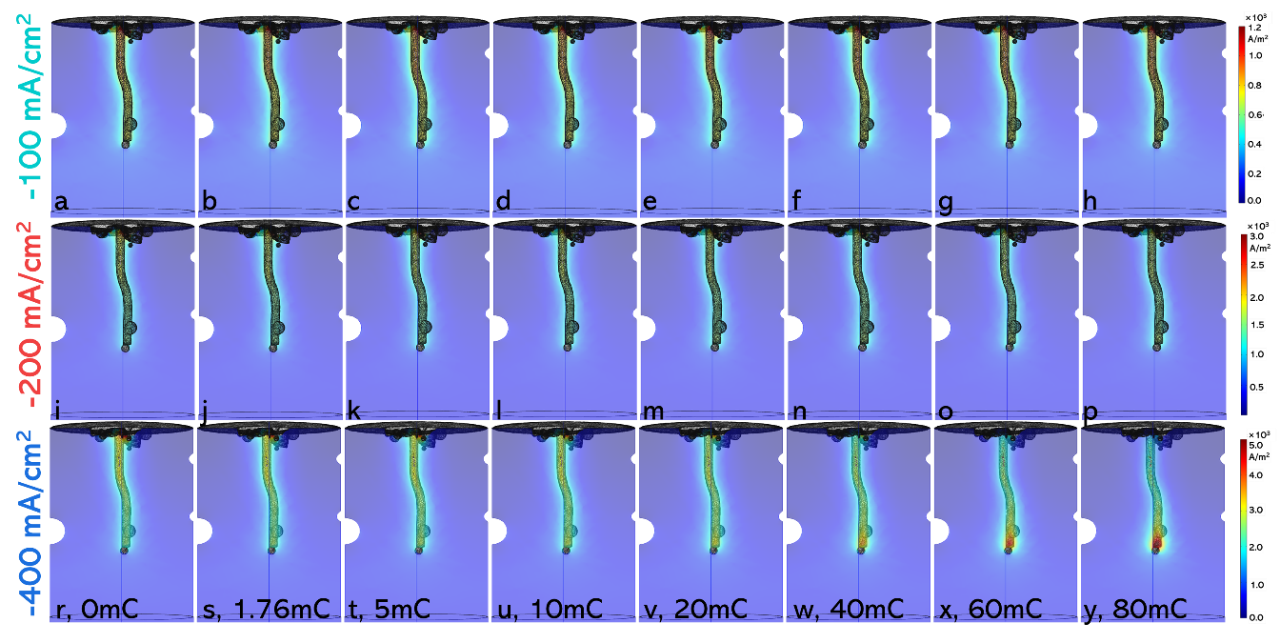


**Fig. S7**. The variations of the simulated current density with the electric charge transferred during the electrolysis process at -100 mA/cm^2^, -200 mA/cm^2^, and -400 mA/cm^2^ by Multiphysics field simulation.

**Fig. S8**. The variations of the simulated voltage with the electric charge transferred during the electrolysis process at -100 mA/cm^2^, -200 mA/cm^2^, and -400 mA/cm^2^ by Multiphysics field simulation.


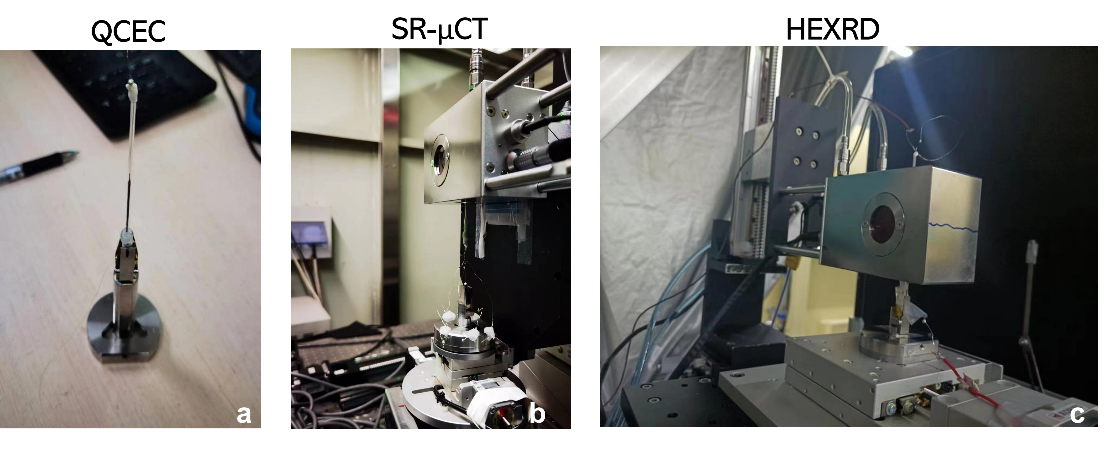


**Fig. S9.** Photographs of (a) QCEC mounted on the holder, (b) setups of operando SR-µCT experiment on the 4W1A beamline, BSRF, and (c) setups of operando HEXRD experiment on the BL13SSW beamline, SSRF.


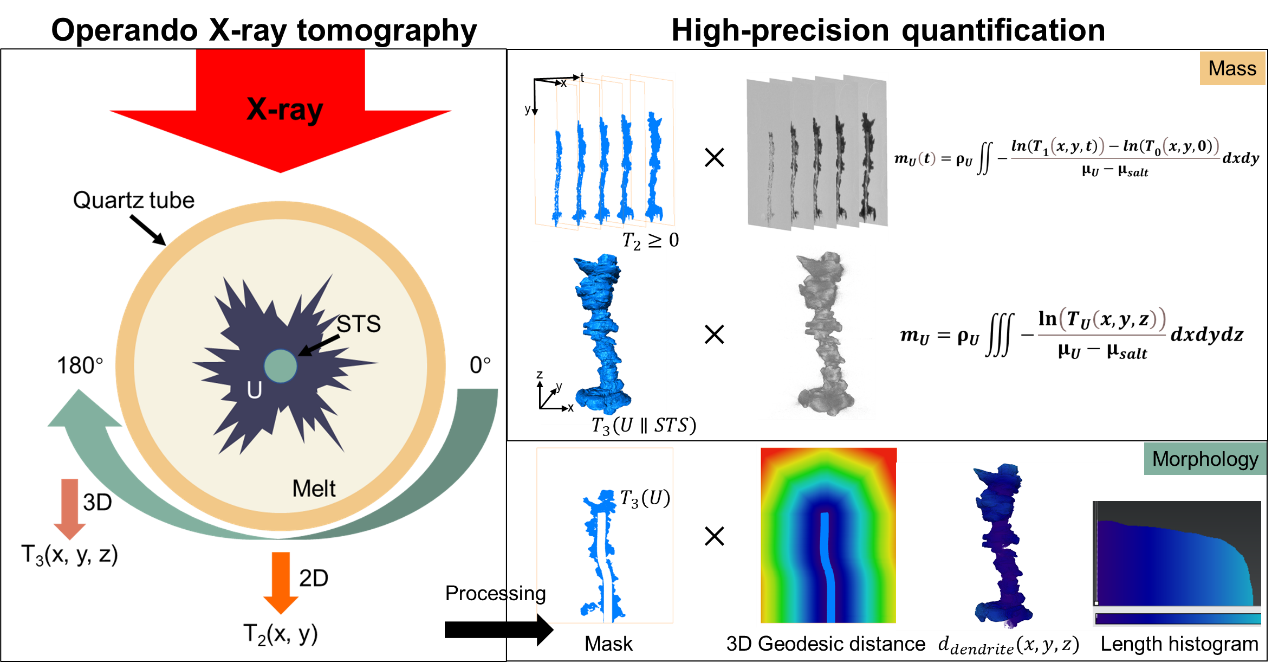


**Fig. S10.** Scheme of the transmission of X-rays through the operando electrolytic cell to collect the time-dependent 2D data and the 3D data and the illustration of the 2D and 3D data processing for visualization and quantification.

**Table S1.** Calculated parameters based on 3D reconstructed data.

| Initial current density (mA/cm^2^) | Surface area (um^2^) | Volume (um^3^) |  | Volume-specific area (um^2^/um^3^) | Normalized volume fraction  (%) |  | Uranium mass^#^  (mg) |
| --- | --- | --- | --- | --- | --- | --- | --- |
| -100 | 9.44×10^5^ | 8.76×10^6^ |  | 0.11 | 13.76 |  | 6.27×10^2^ |
| -200 | 1.47×10^6^ | 7.84×10^6^ |  | 0.19 | 3.76 |  | 6.41×10^2^ |
| -400 | 2.56×10^6^ | 1.22×10^7^ |  | 0.26 | 2.40 |  | 6.69×10^2^ |

^#^The theoretical mass of 80mC is 6.58×10^2^ mg.
